# The Furan Fatty Acids 11M5 and 11D5 Can Act as Activators of Human Peroxisome Proliferator‐Activated Receptor Gamma

**DOI:** 10.1002/mnfr.70309

**Published:** 2025-11-07

**Authors:** Jonas Pospiech, Ayelet Caspi, Walter Vetter, Zohar Kerem, Jan Frank, Thomas A. Kufer

**Affiliations:** ^1^ Institute of Nutritional Medicine University of Hohenheim Stuttgart Germany; ^2^ Department of Food Biofunctionality Institute of Nutritional Sciences University of Hohenheim Stuttgart Germany; ^3^ Robert H. Smith Faculty of Agriculture Food and Environment The Hebrew University of Jerusalem Rehovot Israel; ^4^ Institute of Food Chemistry (170b) University of Hohenheim Stuttgart Germany

**Keywords:** cellular activity, nuclear receptors, pregnane X receptor, retinoid X receptor, transcription regulation

## Abstract

Furan fatty acids (FuFA) are a minor class of fatty acids in food that are characterized by a furan ring within the alkyl chain. Furan fatty acids have strong antioxidant properties but their biological functions remain largely elusive. Using molecular docking combined with biomolecular validation, we investigated the regulatory activities of the key furan fatty acids 9M5, 11M5, and 11D5 on human nuclear receptors, including PPARγ, LXR, PXR, FXR, and HNF4α. Using computational methods, 11M5 and 11D5 and to a lesser extend 9M5 were predicted to bind to PPARγ. The activation of both PPARγ1 and PPARγ2 was confirmed in human HEK293T cells and structure‐activity experiments revealed that this was dependent on the furan fatty acid backbone. In summary, our data provide novel insights into the biological activities of furan fatty acids in human cells and indicate that activation of peroxisome proliferator‐activated receptor gamma underlies their beneficial health effects. These findings establish a clear mechanistic basis, supported by the inactivity of related compounds, and we are confident that future expanded studies will further confirm this mechanism.

## Graphical abstract

Furan fatty acids are abundant in food such as fatty fish. Here we show that some of these can bind to and act as activators of the nuclear receptors, peroxisome proliferator‐activated receptor gamma (PPAR‐gamma) and retinoid X receptor (RXR), in human cells.

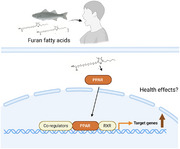

**Abbreviations**
11D5: 11‐(3,4‐dimethyl‐pentylfuran‐2‐yl)‐undecanoic acid
11M5: 11‐(3‐methyl‐5‐pentylfuran‐2‐yl)‐undecanoic acid
9M5: 9‐(3‐methyl‐5‐pentylfuran‐2‐yl)‐nonanoic acid
BSA: bovine serum albumin
DMEM: Dulbecco's Modified Eagle's Medium
FXR: farnesoid X receptor
GC/MS: gas chromatography with mass spectrometry
HNF4α: hepatocyte nuclear factor 4 α
LXR: liver X receptor
PPAR: peroxisome proliferator‐activated receptor
PXR: pregnane X receptor
RXR: retinoid X receptor
SC: solvent control

## 1 Introduction

**FIGURE 1:**
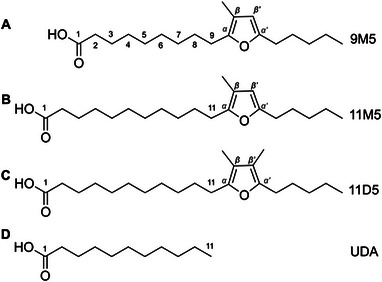
Chemical structures of the furan fatty acids. 9‐(3‐methyl‐5‐pentylfuran‐2‐yl)‐nonanoic acid (A), 11‐(3‐methyl‐5‐pentylfuran‐2‐yl)‐undecanoic acid (B), and 11‐(3,4‐dimethyl‐pentylfuran‐2‐yl)‐undecanoic acid (C) as well as the saturated fatty acid undecanoic acid (undecanoic acid, 11:0) (D). The furan fatty acid nomenclature (A–C) of Vetter and Wendlinger is used. [8]

Nuclear receptors are a large and diverse family of mammalian ligand‐inducible transcription factors that modulate target gene expression in numerous pathways, regulating physiological processes, such as development, reproduction, and metabolism [1]. Upon ligand‐binding, they form homodimers or heterodimers with the retinoid X receptor (RXR) that subsequently bind to specific DNA sites causing transcriptional regulation of target genes [2]. Physiologically relevant endogenous ligands of nuclear receptors include steroid hormones, retinoids, thyroid hormones, and vitamin D_3_ [3]. In addition to endogenous ligands, activation of nuclear receptors can be modulated by compounds present in food. For example, docosahexaenoic acid and eicosapentaenoic acid, two major polyunsaturated fatty acids, can activate members of the peroxisome proliferator‐activated receptor (PPAR) family [4, 5]. Moreover, the regular consumption of docosahexaenoic acid and eicosapentaenoic acid has been associated with a reduced risk of obesity, metabolic syndrome, and coronary heart disease mortality [6, 7]. In addition to these well described major fatty acids, fish contains minor fatty acid classes, such as the furan fatty acids (FuFA) that recently were proposed to contribute to the health beneficial biological effects of fish oil in humans [8]. The basic chemical structure of heterocyclic FuFA includes a furan ring with a carboxyalkyl chain in α‐position and an alkyl chain in α’‐position. The β‐ and β’‐positions are usually substituted with one or two methyl groups. The nomenclature for FuFA recently proposed by Vetter et al. will be used in this work [8]. The length of the carboxyalkyl chain is represented by a number, followed by a letter for the methyl substituent at the β‐ and β’‐position of the furan moiety (either “D” for a dimethyl‐, “M” for a methyl‐, or “F” for an unsubstituted FuFA) and at the last position the length of the alkyl moiety is given as a number. In this nomenclature, the FuFA with the complex chemical name 9‐(3‐methyl‐5‐pentylfuran‐2‐yl)‐nonanoic acid is termed 9M5 (Figure 1).

Due to their electron‐rich furan ring, FuFA act as radical scavengers and antioxidants [9, 10], functions that might contribute to the beneficial effects of fish consumption and fish oil intake that had been previously attributed to docosahexaenoic acid and eicosapentaenoic acid [10]. Due to technical difficulties in synthesizing and isolating these fatty acids, the biological activities of FuFA still remain enigmatic and only a few biological effects are hitherto experimentally documented. Lauvai et al., for example, demonstrated that 9M5 can act as a ligand for PPARγ in COS‐7 cells, leading to increased PPARγ and adiponectin expression [11].

In the present study, we employed computational tools to address our research question, leveraging their predictive capabilities to guide subsequent biological investigations. Utilizing at least two complementary computational approaches enhanced the reliability of biochemical character predictions, as each method offers unique insights and mitigates individual limitations. Accordingly, we applied both molecular docking and MM‐GBSA (Molecular Mechanics–Generalized Born Surface Area) calculations to evaluate ligand–receptor interactions [12, 13].

Molecular docking, particularly using the Glide module (Schrödinger), predicts the preferred binding orientations of small molecules within a protein target and provides an initial estimate of interaction strength through the GlideScore (gScore). This empirical scoring function accounts for factors such as hydrogen bonding, hydrophobic interactions, van der Waals forces, and desolvation penalties. To refine these predictions, we complemented docking with MM‐GBSA calculations, which offer a more thermodynamically rigorous estimate of binding free energy by combining molecular mechanics energies with solvation effects. This approach has been shown to improve the correlation between computational predictions and experimental results, enhancing the accuracy of binding affinity estimations. Based on these in silico studies, we conducted cell biological analyses in vitro to investigate the potential of the three key FuFA found in food, 9M5, 11M5, and 11D5, as regulators of the human nuclear receptors PPARγ, LXR, PXR, FXR, and HNF4α.

## 2 Materials and Methods

### 2.1 Molecular Docking

Molecular docking and MM‐GBSA (Molecular Mechanics Generalized Born Surface Area) calculations were performed using the Maestro (Schrödinger, USA) to assess the binding affinities and interaction profiles between selected ligands and nuclear receptor targets. High‐resolution crystal structures of four nuclear receptor ligand‐binding domains were obtained from the Protein Data Bank: HNF4α (PDB code 1M7W), the Farnesoid X Receptor (FXR) (PDB code 3FLI), PPARγ (PDB code 3NOA), and the Pregnane X Receptor (PXR) (PDB code 6P2B). Each protein structure was processed using the Protein Preparation Wizard, which included the assignment of bond orders, addition of hydrogens, optimization of hydrogen‐bonding networks, and restrained minimization using the OPLS4 force field.

Ligands were prepared using LigPrep at a physiological pH of 7.0 ± 0.2, with generation of possible ionization states and stereoisomers where applicable. Docking simulations were carried out using the Glide module in Extra Precision (XP) mode to accurately predict binding poses within the receptor binding sites. Following docking, MM‐GBSA binding free energy calculations were performed using the Prime module, providing a more refined estimation of ligand binding affinities by incorporating solvation effects and molecular mechanics energies. These computational steps enabled a comparative evaluation of ligand–receptor interactions across the studied nuclear receptors [14].

### 2.2 Plasmids and Reagents

The FuFA 11D5 and 11M5 were purchased from Cayman‐Chemical (Ann Arbor, MI, USA). 9M5 was isolated by Franziska Müller (University of Hohenheim, Institute of Food Chemistry, 170b, Germany), using the protocol of Müller et al. [15].

The plasmids pcD‐huFXR α2, pcD‐huHNF4α, pcD‐huLXRα, pcD‐huLXRβ, pcD‐huPXR, pcDNA3‐hPPARγ1, pcDNA3‐hPPARγ2, pcGL3‐CYP3A4(‐7830/Δ7208‐364), pGL3‐(IR1)_2_TK, pGL3‐bisHNFα RE‐TK(‐105), and pGL3‐TK (SMPDL3A‐LXRE) were kindly provided by Dr. Oliver Burk (Dr. Magarete Fischer‐Bosch‐Institute of Clinical Pharmacology, Stuttgart, Germany). The plasmid PPRE‐X3‐TK‐luc was kindly provided by Monika Schumacher (University of Hohenheim, Institute of Nutritional Sciences, Stuttgart, Germany).

### 2.3 Cell Culture

The human embryonic kidney cell line HEK293T was purchased from ATCC. The human hepatocellular carcinoma cell line HepG2 was kindly provided by Prof. Lutz Graeve (University of Hohenheim, Institute of Nutritional Sciences, Stuttgart, Germany). Cells were grown in Dulbecco's Modified Eagle's Medium (DMEM) high glucose (4.5 g/L D‐glucose, Thermo Fisher Scientific, Waltham, MA, USA), supplemented with 10% heat‐inactivated fetal calf serum (PAN‐Biotech, Aidenbach, Germany) and 1% penicillin/streptomycin (Thermo Fisher Scientific) at 37°C in a 5% CO_2_ atmosphere. Cells were continuously tested for the absence of mycoplasma by PCR.

### 2.4 Stimulation With Fatty Acids

9M5 was dissolved in *n*‐hexane. 11D5, 11M5 and undecanoic acid (11:0; a carboxylic acid without a furan ring) were dissolved in 100% ethanol. To protect fatty acids from oxidation, the prepared stock solutions (28–50 mmol/L) were stored under nitrogen gas at −80°C. Prior to addition to the cells, fatty acids were complexed with bovine serum albumin (BSA) as described by Chavez et al. [16] with minor changes. Fatty acids were diluted 1:20 in 10% BSA in phosphate‐buffered saline (PBS) and incubated at 55°C for 10 min under constant shaking.

### 2.5 9M5 Uptake in HepG2 Cells

Cells were incubated for 24 h at 37°C in a 5% CO_2_ atmosphere in 10 mL DMEM high glucose with 9M5 in concentrations of 1.0, 2.5 and 5 µg/mL. After incubation, the medium was removed and frozen at −80°C. The cells were harvested and centrifuged for 5 min at 180 × *g*. The supernatant was discarded, and the cell pellet frozen at −80°C until further testing. A total of 2 mL of methanol with 1% H_2_SO_4_ was added to the sample (cell pellet or medium) and the sample was sonicated three times for 5 min each and subsequently heated to 80°C for 2 h. The sample was placed on ice and 2 mL *n*‐hexane and 1 mL saturated NaCl solution were added, and the organic phase was used for gas chromatography with mass spectrometry analysis.

### 2.6 Quantitative Analysis of Furan Fatty Acids

Quantification of FuFA by gas chromatography with mass spectrometry using a 6890/5973 N system (Agilent, Waldbronn, Germany) in full scan mode (*m/z* 50‐650) was performed after conversion into the corresponding methyl esters. Additional measurements were performed in selected ion monitoring mode. The injector and detector temperatures were set to 250°C and 280°C. Helium (purity 99.999 %) was used as the carrier gas at a constant flow rate of 1 mL/min. The separation was performed using a 30 m × 0.25 mm inner diameter capillary column coated with 0.25 µM of 5% phenyl‐ and 95% methyl‐polysiloxane (HP‐5MS UI, Agilent, Waldbronn, Germany). The GC oven was programmed as follows: after 1 min at 60°C, the temperature was ramped at 13°C/min to 180°C, at 3°C/min to 250°C, and at 20°C/min to 300°C, which was held for 5 min [17]. The analysis was carried out using the mass‐to‐charge ratios of *m/z* 165 (9M5, 11M5) and *m/z* 179 (11D5). The concentrations were determined using an external standard (9M5 ethyl ester) and normalized to the number of cells.

### 2.7 Luciferase Reporter Assay

HEK293T cells (30 000 cells/well) were seeded in 96‐well plates and incubated for 1 h at 37°C in 5% CO_2_ and transiently transfected using XtremeGene9 from Roche (Mannheim, Germany). β‐galactosidase plasmid (8.6 ng), 20 ng of the receptor‐specific luciferase reporter plasmid and 1 ng of receptor‐specific expression plasmid were used for transfection, using a total DNA amount 113.6 ng plasmid per well, adjusted by adding pcDNA. Cells were incubated for 1 h at 37°C in a 5% CO_2_ atmosphere before incubation with FuFA. Depending on the FuFA used, either *n*‐hexane or ethanol at a volume identical to that of the solution with the highest concentration of the respective FuFa were used as solvent control. The fatty acid concentrations were set as follows: 0, 1, 5, 10, and 20 µmol/L. After treatment, the cells were incubated for 21–24 h at 37°C in 5% CO_2_. After incubation, the cells were lysed, and luciferase activity was quantified using a Perkin Elmer EnSpire luminometer (Waltham, MA, USA). To normalize the readout to the number of transiently transfected cells, the β‐galactosidase activity was read at 405 nm (620 nm as reference) by o‐nitrophenyl‐β‐D‐galactopyranoside assay.

### 2.8 Statistical Analyses

Data were analyzed using GraphPad Prism 8 for macOS (Version 8.4.3, San Diego, CA, USA). Differences between solvent control and treatment means were calculated using the one‐way‐ANOVA, followed by Dunnett's multiple comparison test (each experiment was performed twice with three independent replicates (wells) each; *n* = 6). The level of significance was set at *p* < 0.05.

## 3 Results

### 3.1 Molecular Docking Identifies Nuclear Receptors as Potential Binding Targets of Furan Fatty Acids

The binding affinities of the selected furan fatty acids (FuFA) 9M5, 11M5, or 11D5 to the four human nuclear receptors were assessed using MM‐GBSA calculations following XP docking. We focused our analysis on the most prominent human nuclear receptors that are involved in the regulation of metabolic processes and cell signaling, namely peroxisome proliferator‐activated receptors (PPAR), liver X receptors (LXR), pregnane X receptor (PXR), farnesoid X receptor (FXR), and hepatocyte nuclear factor 4 (HNF4). 11M5 exhibited the most favorable binding energy with PPARγ (–55.56 kcal/mol; Table 1), indicating high predicted binding affinity. 9M5 showed best binding to FXR (–49.7 kcal/mol; Table 1), while 11D5 was predicted to best bind to PXR (–51.00 kcal/mol; Table 1). No MM‐GBSA result was obtained for ligand 11M5 with HNFα due to a failed docking pose (indicated as “X”; Table 1).

**TABLE 1:** MM‐GBSA binding free energies (kcal/mol) and Glide docking scores (gScore) for the three ligands (9M5, 11M5, 11D5) across four nuclear receptors: HNFα, FXR, PXR, and PPARγ. The most favorable value for each receptor is highlighted in bold. X: Failed docking pose.

	Receptor	9M5	11M5	11D5
MM‐GBSA	HNFα	**−43.89**	X	−25.66
	PPARgamma	−42.35	**−55.56**	−53.00
	PXR	−44.74	−36.00	**−51.00**
	FXR	**−49.70**	−47.11	−38.97
gScore	HNFα	**−7.88**	−5.51	−6.66
	PPARgamma	−7.98	−8.57	**−9.32**
	PXR	**−8.07**	−7.56	−8.07
	FXR	−7.30	**−7.80**	−7.73

**FIGURE 2:**
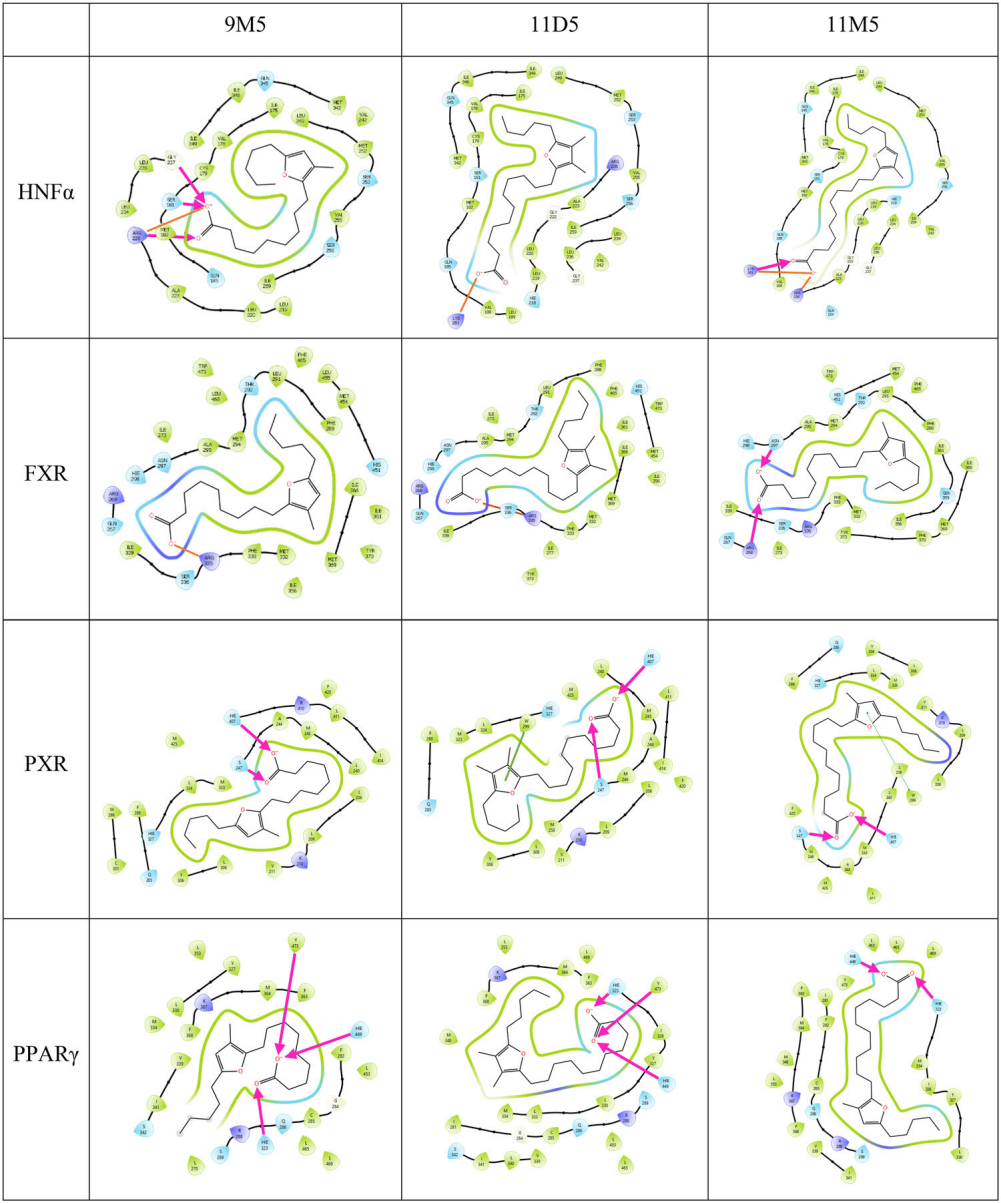
2D interaction diagrams of FuFAs with the ligand‐binding domains of HNFα, FXR, PXR, and PPARγ, as generated from docking simulations. Each panel shows the interaction profile of one ligand (9M5, 11D5, or 11M5) with a specific receptor, highlighting hydrogen bonds, hydrophobic contacts, and *π*–*π* stacking interactions.

The 2D interaction diagrams (Figure 2) illustrate the specific molecular interactions formed between each ligand and the receptor binding sites. These diagrams highlight the presence of multiple hydrophobic contacts and hydrogen bonds, particularly in the high‐affinity complexes. For example, 11M5 formed hydrogen bonds with residues in the PPARγ binding site, supporting its strong MM‐GBSA score. Similarly, 9M5 demonstrated a well‐defined interaction pattern within the FXR binding domain, correlating with its favorable binding energy. In contrast, 11D5 exhibited less extensive interactions in several complexes.

### 3.2 Cellular Activation of Human Nuclear Receptors by Furan Fatty Acids

**FIGURE 3:**
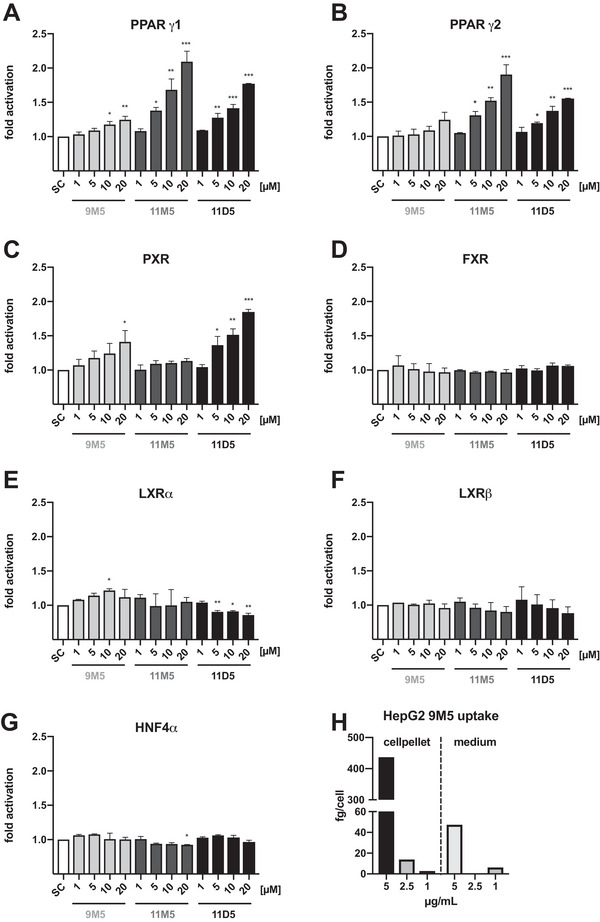
Effects of 9M5, 11M5, and 11D5 on nuclear receptor activity. Activity of the furan fatty acids was tested for (A) PPARγ1, (B) PPARγ2, (C) pregnane X receptor (PXR), (D) FXR, (E) LXRα, (F) LXRβ, and (G) HNF4α. HEK293T cells were transfected with the indicated reporter plasmids and incubated with either 9M5, 11M5, or 11D5 at concentrations of 0–20 µmol/L. BSA and *n*‐hexane (9M5) or ethanol (11M5 and 11D5) was used as solvent control (SC). Data reflect relative light units normalized to β‐galactosidase expression. Bars represent arithmetic means + standard deviation (*n* = 6; duplicate experiments with three replicates each). Significant differences in fold‐activation compared to SC were calculated using one‐way‐ANOVA, followed by Dunnett's multiple comparison test * (*p* < 0.05), ** (*p* < 0.01), *** (*p* < 0.001). (H) Uptake of the furan fatty acid 9M5 into HepG2 cells after 24 h incubation. Cells were treated with 1.0, 2.5, or 5 µg/L 9M5 for 24 h and 9M5 uptake was determined by acid esterification using gas chromatography with mass spectrometry (GC/MS).

To confirm the potential of FuFA to modulate nuclear receptor signaling, we applied reporter‐gene assays for a panel of human nuclear receptors using transiently transfected HEK293T cells. Assay conditions for each receptor were optimized by titrating different concentrations of plasmids and agonists. The uptake of FuFA into cells, which is a prerequisite for the activation of intracellular receptors, was verified using 9M5 and human HepG2 cells (Figure 3H).

Cells were incubated for 24 h with either 9M5, 11M5 or 11D5 in concentrations ranging from 1 to 20 µmol/L and receptor activities were measured using luciferase activity as reporter gene. A dose‐dependent activation by FuFA was observed for both PPARγ isoforms and PXR (Figure 3A–C). None of the tested FuFA activated LXR, FXR, or HNF4α in our system, 11D5 even significantly inhibited the LXRα reporter (Figure 3D–G). Both PPARγ isoforms were activated by 9M5 stimulation, which was statistically significant for PPARγ1 at high concentrations (Figure 3A,B). The highest induction of PPARγ was obtained with 11M5, which led to a 2‐fold increase relative to the respective solvent control (*p* < 0.05), which is also in line with the docking data that showed the lowest free energy data for all tested combinations. Incubation of HEK293T cells with 11D5 at concentrations of 5 µmol/L or higher significantly and dose‐dependently increased PPARγ activity (up to 1.8‐fold for PPARγ1 and 1.6‐fold for PPARγ2) relative to the respective solvent control. PXR was also dose‐dependently activated by 9M5 and 11D5 (Figure 3C), with 9M5 inducing PXR approximately 1.5‐fold (*p* < 0.05) and 11D5 nearly 2‐fold at the highest concentration of 20 µmol/L.

11D5 appears more potent than 9M5 and activated PXR at concentrations as low as 5 µmol/L, whereas 11M5 did not modulate PXR activity. These data also suggest, consistent with our docking analysis, that 11D5 followed by 9M5 are the better PXR binders here.

Taken together, these data establish 9M5, 11M5, and 11D5 as activators of PPARγ, and 9M5 and 11D5 as PXR agonists in cells. Combined with the docking data, these findings identify 11M5 and 11D5 as relevant PPARγ modulators and highlight 11D5 as a novel PXR agonist. Our data strongly support direct binding of FuFa to nuclear receptors, even though molecular evidence based on targeted mutation of binding sites is lacking. This compelling conclusion thus warrants future exploration using additional mechanistic approaches and models to fully define its structural basis.

### 3.3 The Carboxyalkyl Chain of 11M5 Is Not Sufficient for the Activation of PPARγ and PXR

**FIGURE 4:**
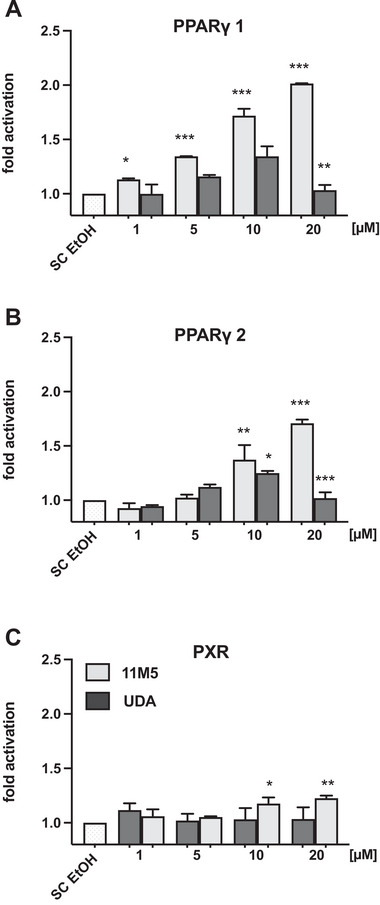
Effects of undecanoic acid (11:0) and 11M5 on PPARγ and PXR activity. HEK293T cells were transfected with the indicated receptor plasmids. Cells were incubated with either 11M5 or undecanoic acid at concentrations of 0–20 µmol/L. BSA and ethanol served as solvent control (SC). (A) PPARγ1, (B) PPARγ2, and (C) PXR activity is shown as fold‐induction over SC. Bars represent arithmetic means + standard deviation (*n* = 6; duplicate experiments with three replicates each). Significant differences in fold‐induction compared to SC were calculated using one‐way‐ANOVA, followed by Dunnett's multiple comparison test * (*p* < 0.05), ** (*p* < 0.01), *** (*p* < 0.001).

As shown above, 11M5 was the strongest inducer and best binder of PPARγ. As PPARγ is activated by numerous conventional fatty acids [5], we hypothesized that the carboxyalkyl chain of 11M5 and 11D5 might be sufficient for the activation. To this end, HEK293T cells were transfected and incubated with either 11M5 or undecanoic acid, a saturated fatty acid containing the same carboxyalkyl chain length (11 carbon atoms) as 11M5 prior to the furan moiety (Figure 1D). Reporter gene assays were carried out and the receptor activity was evaluated. As shown before (Figure 3A,B), an increased dose‐dependent receptor activation was obtained for both PPARγ isoforms upon treatment with 11M5 (Figure 4A,B). This increase was significant for all concentrations tested and achieved a 2‐fold upregulation at high concentrations relative to the solvent control (*p* < 0.0001; Figure 4A). In contrast, when incubating cells with undecanoic acid, receptor activity was increased to a maximum 1.35‐fold for PPARγ1 (*p* < 0.05) and 1.25‐fold for PPARγ2 (*p* < 0.05) compared to their respective controls. For PXR, a slightly increased dose‐dependent activation was observed upon 11M5 treatment (about 1.2‐fold relative to the SC (*p* < 0.05)), while undecanoic acid stimulation had no detectable effect on PXR signal intensity (Figure 4C).

In summary, our data showed that PPARγ signaling can be modulated by furan fatty acids. This effect was not dependent on the carboxyalkyl chain itself but seems to be a unique feature of the furan fatty acid backbone. This is supported by the fact that PXR was not activated by undecanoic acid, but by 11M5 and 9M5, even though the latter has lower activity than 11M5.

## 4 Discussion

Effects of the most prominent dietary fatty acids on nuclear receptor activation are well documented [4, 5, 18]. However, little is known about the effects of minor fatty acids, in particular furan fatty acids, on nuclear receptor signaling. Using a screen to systematically investigate the activities of selected furan fatty acids on the most relevant human nuclear receptors, our work provides first insights into the biological functions of the furan fatty acids 9M5, 11M5, and 11D5.

We started with a docking analysis that suggested the highest binding affinities for 11M5 and 11D5 with PPARγ. Our results demonstrate that MM‐GBSA, unlike GlideScore (gScore), provided a good prediction of ligand–receptor binding affinity that reflects well the data from our biological assays. This aligns with Lyne et al., who showed that GlideScore, while effective for pose prediction, often fails to correlate with experimental data [19]. MM‐GBSA, which incorporates solvation and surface area energy terms, provided more accurate binding energy estimations and is recommended for guiding compound prioritization in structure‐based drug design.

Using cell‐based reporter assays, we validated 11M5 and 11D5 as activators of PPARγ, which is well‐known as the master regulator for adipogenesis. 9M5 also showed some PPARγ‐inducing activity, albeit lower compared to 11M5 and 11D5. This is in line with previous work from Lauvai et al. who analyzed 9M5 activity in the African green monkey kidney cell line COS‐7 and observed an induction of PPARγ signaling [11]. Our study corroborates these results in another cellular model. Moreover, we expand on this previous work and show that 11M5 and 11D5, which have a longer carboxyalkyl chain before the furan ring compared to 9M5, showed even higher transcriptional activity for PPARγ than 9M5 (Figure 3A,B). The observation that undecanoic acid, which has the same carboxyalkyl chain length but lacks the furan moiety, did not activate PPARγ strongly suggests that additional characteristic features of furan fatty acids are required for PPARγ activation. The PPARγ ligand‐binding site is a large hydrophobic t‐shaped cavity located at the C‐terminal helix and β‐sheet lying between helices H3 and H6. The amino acids His^323^, His^449^, Ser^289^, and Tyr^473^ are located in the PPARγ‐ligand binding domain and are crucial for PPARγ activation [20]. The formation of hydrogen bonds of these amino acids in the ligand binding domain with polar functional groups, such as carbonyl or carboxyl oxygen atoms of potential agonists, results in a conformational change of the ligand‐binding domain‐activation function‐2 region, which is pivotal for co‐activator recruitment and interaction [20]. Rosiglitazone is a known agonist for PPARγ containing a benzene‐ and pyridine ring as well as a thiazolidinedione head group. Its functional thiazolidinedione head group forms primary hydrogen bonds with His^323^ and His^449^ residues as well as secondary hydrogen bonds with Tyr^473^ with His^323^ and Lys^367^ with the negatively charged nitrogen of the thiazolidinedione head group causing PPARγ activation [21]. Natural carboxylic acids, such as fatty acids, seem to be able to form key interactions with His^323^ and His^449^ residues [21]. Due to their polar furan ring, furan fatty acids might form additional hydrogen bonds that lead to higher binding affinities and thus a stronger PPARγ activation, as suggested by the docking analysis (Figure 2). The slightly higher activation of both PPARγ isoforms by 11M5 compared to 11D5 might be explained by its second methyl group in β’‐position, which that changes interaction with the residues located in the PPARγ‐ligand binding domain (Figure 2). However, while this interpretation is currently speculative, it represents a strong and novel conclusion that invites future studies, such as site‐directed mutagenesis and complementary structural or biophysical approaches, to fully substantiate and extend these findings.

We furthermore observed activation of the xenobiotic receptor PXR by furan fatty acids. PXR, in contrast to PPARγ, showed specificity toward 11D5 and was also weakly activated by 9M5 (Figure 2C). Because, in our experiments, 11M5 induced significantly lower PXR activity compared to 11D5 (Figure 2C), hydrophobic residues, such as methyl groups, as well as their position might be crucial for PXR activation. Future and more detailed experiments are warranted to elucidate the structural requirements for activation of both nuclear receptors.

As furan fatty acids are easily degraded during storage, the concentrations required for nuclear receptor activation in the present experiments should be interpreted with caution. It was previously reported that sidechain‐shortened metabolites, namely 3M5, can be detected in the supernatant of cells incubated with 9M5 [11]. Hence, we cannot exclude that the observed effects might be caused by degradation products and/or metabolites of the furan fatty acids. Because the present experiments are not able to answer this question, further experiments aimed at elucidating the cellular metabolism of furan fatty acids as well as interactions of other metabolites or degradation products formed with these nuclear receptors are warranted.

## 5 Conclusion

In conclusion, our findings suggest that the furan fatty acids 9M5, 11M5, and 11D5 act as potential ligands for PPARγ and PXR in vitro. Molecular docking and in vitro biochemical activity thereby highlight 11M5 and 11D5 as the FuFA with the highest activity. The underlying mechanisms and the implications for human health warrant further investigation.

## Funding

The authors have nothing to report.

## Conflicts of Interest

The authors declare no conflicts of interest.

## Data Availability

The original raw data supporting the findings of this article are available from the corresponding author upon reasonable request.

## References

[1] H. Gronemeyer , J. A. Gustafsson , and V. Laudet , “Principles for Modulation of the Nuclear Receptor Superfamily,” Nature Reviews Drug Discovery 3 (2004): 950–964. 15520817 10.1038/nrd1551

[2] D. J. Mangelsdorf , C. Thummel , M. Beato , et al., “The Nuclear Receptor Superfamily: The Second Decade,” Cell 83 (1995): 835–839. 8521507 10.1016/0092-8674(95)90199-x PMC6159888

[3] E. R. Weikum , X. Liu , and E. A. Ortlund , “The Nuclear Receptor Superfamily: A Structural Perspective,” Protein Science 27 (2018): 1876–1892. 30109749 10.1002/pro.3496 PMC6201731

[4] S. A. Kliewer , S. S. Sundseth , S. A. Jones , et al., “Fatty Acids and Eicosanoids Regulate Gene Expression Through Direct Interactions With Peroxisome Proliferator‐Activated Receptors α and γ,” Proceedings of the National Academy of Sciences of the United States of America 94 (1997): 4318–4323. 9113987 10.1073/pnas.94.9.4318 PMC20720

[5] G. Krey , O. Braissant , F. L'Horset , et al., “Fatty Acids, Eicosanoids, and Hypolipidemic Agents Identified as Ligands of Peroxisome Proliferator‐Activated Receptors by Coactivator‐Dependent Receptor Ligand Assay,” Molecular Endocrinology 11 (1997): 779–791. 9171241 10.1210/mend.11.6.0007

[6] A. Leaf and P. C. Weber , “Cardiovascular Effects of n‐3 Fatty Acids,” New England Journal of Medicine 318 (1988): 549–557. 3277056 10.1056/NEJM198803033180905

[7] M. L. Burr , A. M. Fehily , J. F. Gilbert , et al., “Effects of Changes in Fat, Fish, and Fibre Intakes on Death and Myocardial Reinfarction: Diet and Reinfarction Trial (DART),” Lancet 2 (1989): 757–761. 2571009 10.1016/s0140-6736(89)90828-3

[8] W. Vetter and C. Wendlinger , “Furan Fatty Acids—Valuable Minor Fatty Acids in Food,” Lipid Technology 25 (2013): 7–10.

[9] R. A. Lemke , A. C. Peterson , E. C. Ziegelhoffer , et al., “Synthesis and Scavenging Role of Furan Fatty Acids,” Proceedings of the National Academy of Sciences of the United States of America 111 (2014): E3450–3457. 25092314 10.1073/pnas.1405520111 PMC4143029

[10] G. Spiteller , “Furan Fatty Acids: Occurrence, Synthesis, and Reactions. Are Furan Fatty Acids Responsible for the Cardioprotective Effects of a Fish Diet?,” Lipids 40 (2005): 755–771. 16296395 10.1007/s11745-005-1438-5

[11] J. Lauvai , A. K. Becker , K. Lehnert , et al., “The Furan Fatty Acid 9M5 Acts as a Partial Ligand to Peroxisome Proliferator‐Activated Receptor *Gamma* and Enhances Adipogenesis in 3T3‐L1 Preadipocytes,” Lipids 54 (2019): 277–288. 31087413 10.1002/lipd.12152

[12] T. Hou , J. Wang , Y. Li , and W. Wang , “Assessing the Performance of the Molecular Mechanics/Poisson Boltzmann Surface Area and Molecular Mechanics/Generalized Born Surface Area Methods. II. The Accuracy of Ranking Poses Generated From Docking,” Journal of Computational Chemistry 32 (2011): 866–877. 20949517 10.1002/jcc.21666 PMC3043139

[13] D. Jiang , H. Du , H. Zhao , et al., “Assessing the Performance of MM/PBSA and MM/GBSA Methods. 10. Prediction Reliability of Binding Affinities and Binding Poses for RNA–Ligand Complexes,” Physical Chemistry Chemical Physics 26 (2024): 10323–10335. 38501198 10.1039/d3cp04366e

[14] M. S. Naamneh , T. Momic , M. Klazas , et al., “Structure‐Activity Relationship of Synthetic Linear KTS‐Peptides Containing Meta‐Aminobenzoic Acid as Antagonists of alpha1beta1 Integrin With Anti‐Angiogenic and Melanoma Anti‐Tumor Activities,” Pharmaceuticals (Basel) 17 (2024): 549. 38794120 10.3390/ph17050549 PMC11124490

[15] M. Müller , M. Hogg , K. Ulms , and W. Vetter , “Concentrations, Stability, and Isolation of the Furan Fatty Acid 9‐(3‐Methyl‐5‐pentylfuran‐2‐yl)‐Nonanoic Acid From Disposable Latex Gloves,” Journal of Agricultural and Food Chemistry 65 (2017): 7919–7925. 28817932 10.1021/acs.jafc.7b02444

[16] J. A. Chavez , W. L. Holland , J. Bar , K. Sandhoff , and S. A. Summers , “Acid Ceramidase Overexpression Prevents the Inhibitory Effects of Saturated Fatty Acids on Insulin Signaling,” Journal of Biological Chemistry 280 (2005): 20148–20153. 15774472 10.1074/jbc.M412769200

[17] W. Vetter , K. Ulms , C. Wendlinger , and J. van Rijn , “Novel Non‐Methylated Furan Fatty Acids in Fish From a Zero Discharge Aquaculture System,” NFS Journal 2 (2016): 8–14.

[18] A. Chawla , J. J. Repa , R. M. Evans , and D. J. Mangelsdorf , “Nuclear Receptors and Lipid Physiology: Opening the X‐Files,” Science 294 (2001): 1866–1870. 11729302 10.1126/science.294.5548.1866

[19] P. D. Lyne , M. L. Lamb , and J. C. Saeh , “Accurate Prediction of the Relative Potencies of Members of a Series of Kinase Inhibitors Using Molecular Docking and MM‐GBSA Scoring,” Journal of Medicinal Chemistry 49 (2006): 4805–4808. 16884290 10.1021/jm060522a

[20] G. Pochetti , C. Godio , N. Mitro , et al., “Insights Into the Mechanism of Partial Agonism: Crystal Structures of the Peroxisome Proliferator‐Activated Receptor Gamma Ligand‐Binding Domain in the Complex With Two Enantiomeric Ligands,” Journal of Biological Chemistry 282 (2007): 17314–17324. 17403688 10.1074/jbc.M702316200

[21] R. T. Nolte , G. B. Wisely , S. Westin , et al., “Ligand Binding and Co‐Activator Assembly of the Peroxisome Proliferator‐Activated Receptor‐γ,” Nature 395 (1998): 137–143. 9744270 10.1038/25931

